# Correspondence

**Published:** 1869-09

**Authors:** 


### Correspondence.

*To the Editor of the Buffalo Medical and Surgical Journal:*

DEAR SIR,—Believing that every important advance in Uterine Surgery should be published to the profession, I send you the enclosed note from Dr. Atlee, of Philadelphia. Regretting that my own observation does not induce me to hope for the discussion of large fibrous growths by the means indicated by the celebrated gentleman, and hoping that future observation may show me in error, I will not indulge in further comment.

I should state that the letter is in reply to one addressed, a few weeks since, to Dr. Atlee, stating that I had a patient of respectability and wealth, who had been informed by some *lay friends* of his that he was in the habit of excising fibroid growths of the uterus. It was stated, in my communication to him, that the patient was forty-four years old, had suffered with this growth for ten or twelve years, that she was now as large as the woman at term, (weight of tumor estimated at twelve or fifteen pounds;) that it was hard in character, and that I could not determine whether it was intramural or only involved the uterus externally, but that it was not within the cavity of the organ; that the patient suffered little in her general health, had no severe hemorrhages or pains, and was anxious to know if he would relieve her of her deformity, and that I wrote at her request.

I give you this remarkable letter precisely as written, and in full, merely italicizing some of the passages, which seem to me extraordinary, and remain,

Ever truly yours,

JAMES P. WHITE.

PHILADELPHIA, Aug. 17, 1869.

MY DEAR SIR,—I rec'd yours of the 16th to-day. Certain forms of Uterine Fibroids I remove by the knife; but in deciding on the propriety of an operation in any case, it is necessary to make a careful personal examination. As the patient, however, is "in pretty good health," and there is "no hemorrhage," the case is not urgent. I would, therefore, advise *medication, which sometimes will disperse these growths. Give her ten grains of muriate of ammonia three times a day;* and also, twice a day, order her to wash the abdomen well with a solution of it, ℥ii to the pint of water. Persist in this treatment for a long time, provided the health remains good and the tumor does not increase. *If convenient I would like to see her.*

Very respectfully yours,

WASHINGTON L. ATLEE,

1480 Arch St.

To J. P. WHITE, M. D.,

Buffalo, N. Y.

Fee, $10.

Why Prof. White should furnish without comment such a communication we are wholly unable to conceive, but publish it mainly from respect to the distinguished Physicians whose opinions it indicates. It could not be possible that Dr. White wanted to know anything of Dr. Atlee's opinions about uterine tumors, their removal or discussion, being himself fully acquainted with the whole field of human knowledge in this department. The simple answer, to satisfy a patient who had heard wonderful things of Dr. Atlee was all that could have been expected. Such a tumor as you describe cannot, with safety or propriety, be removed by operation or otherwise, was all that any rational physician could say. These letters, we suppose, are open for comment, as they are furnished for publication, and are supposed to be for instruction or *warning*. Dr.

Atlee has given a written opinion in a given case, for which he accepts a fee; and certainly, if he advises his *confrere*, and charges for his opinions, the profession are fairly entitled to the advantages.

He advises " medication," as the case is not urgent. This is a very singular suggestion to make, even if made to a patient, but when made to a member of the profession of distinguished attainments in this department, it is astonishing. Medication to remove a fibrous tumor of ten or twelve years duration and of twelve or fifteen pounds weight. A man must have greater faith than that of Moses to smite such a rock with so slim a rod. Whoever knows anything at all about these growths, and anything about the effects of medicines, understands how unspeakably absurd is the proposition. He says: "Which will sometimes disperse these growths." If the Doctor's experience justifies this assertion, he has doubtless been in error in his diagnosis and regarded some inflammatory or suppurative disease, which disappeared spontaneously, as carried off by medication, for certainly *such* tumors as are fibrous in character, of years duration, and of such size, are *never* dispersed by medication. It seems impossible that Dr. Atlee should hold to such opinion; but, if so, we shall hereafter cease to wonder how men of much less education and attainment can believe in the influence of the most attenuated dilutions. If minds of culture can come to such conclusions, we are prepared for delusions far greater and more manifest than have ever yet disgraced the history of medicine and of mankind. In conclusion, he says: "If convenient, I should be happy to see her." We should scarcely think it would pay. Is it possible that the Doctor can entertain the idea of removing this tumor with the entire uterus, or a part of it?

———:o:———

*To the Editor of the Buffalo Medical and Surgical Journal:*

Dear Sir,—I am led, by some articles which have appeared in your Journal during the last few months, to beg leave to write you a letter of inquiry regarding one of them, particularly, which was published in the July number, by C. C. F. Gay, M. D., of Buffalo, entitled "Puerperal Eclampsia."

The writer states that he "has not sought to enlighten the older members of the profession, but to throw out some hints which may serve as *safe* guidance for those who have but recently entered upon the responsible duties of a laborious and ever harrassing profession." After laboring through several pages of hypothetical pathology, he sums up the treatment as follows: "I believe that, while convulsions last, morphia, gr. 1, is not too much to use hypodermically every half hour."

I am a younger member of this truly harrassing profession, and have encountered but one case of puerperal convulsions, which case ended in recovery, notwithstanding the use of forcible dilatation of the os uteri, chloroform and forceps. I did not then know that gr. 1 of morphia, hypodermically injected, was safe treatment for a young doctor to give, or a patient to take.

Young members of the profession rely upon Medical Journals, more or less, for safe hints, and as a guide to their "prentice hand" in practice; and, in behalf of such, I would ask, do you consider that quantity of morphia, used hypodermically, safe treatment in puerperal convulsions, or in anything else?

The author also states three times in his article, that we are not to force an undilated os uteri. It is quite common, I believe, in cases of rigid, undilatable os uteri, for the first stage of labor to last twenty-four hours or longer. This condition of the uterus may obtain in a case of convulsions. Would it be safe treatment to follow the teaching of the "Buffalo Medical and Surgical Journal," according to Dr. Gay, in such a case, and attend to the convulsions by giving morphia, gr. 1, every half hour while the convulsions lasted and let the uterus alone? Unless the patient were a Chinese opium smoker, or an habitual morphine eater, could one bear it? It seems to me that such therapeutics is more flattering to the druggist than to any physician or medical journal.

Prof. Elliott, of Bellevue Hospital Medical College, says: "at the present day we command the cervix uteri;" and gives elaborate directions how to force an undilated os uteri when required, by sponge tents, Barnes' dilators, and warm water douche. Is dilatation of the os uteri in labor a physiological, vital act of itself, or simply the result of combined forces. However this may be, what harm is there, if circumstances demand it, in aiding the operations of nature by judicious force properly applied from without?

Some of us young doctors may soon have a case of puerperal convulsions. Please state for our guidance in that trying hour, whether we can be justified in giving morphia to the extent mentioned, or attempt in any degree, under any circumstances, to aid the dilatation of the os uteri by mechanical means.

Very respectfully,

ROMAINE J. CURTISS, M. D.,
Angola, New York.

We supposed our readers were by this time aware of the necessity of making some allowances for the expressions of positive and enthusiastic men. We do not hold ourselves personally responsible for the soundness of the doctrines of any of our contributors, though we generally mean in some way to put our attentive readers on guard against error, so that, as a rule, the profession may regard the Journal as a safe practical guide. Our pages, however, are open for the freest expression of opinion; and we hope that no one will infer that we only publish such articles as commend themselves to our personal approval.

In answer to the very pertinent inquiries of our correspondent, we would answer briefly. The question of delivery in case of puerperal convulsions is too important and too extensive to be answered in few words. We could hardly express our opinions without explanations involving a full consideration of the subject. We would advise our young friends desirous of safe guide, to read such recent works as they may have upon obstetrics, where the sentiment repeated by Dr. Gay, that the causes of puerperal eclampsia are to be sought for in a general alteration of the economy and not in the condition of pregnancy, as was formerly supposed, will be found fully explained. Though there is probably some truth in this doctrine, yet the general advice in puerperal eclampsia, to attend to the convulsions and let the uterus alone, may certainly convey error, if by "let the uterus alone," is intended, to in no way interfere with, aid, or hasten the process of labor. In regard to the dose of 1 grain of morphine, subcutaneously, every half hour, being "not too much," we must frankly confess that it *looks large.* We have never tried it, and cannot speak from experience. If we found one patient who could bear it, we should regard it the exception and not the general rule. Our readers must therefore accept the assurance of Dr. Gay, that it is not too much, until future experience shall demonstrate its correctness; but it would seem to us to be a "*leetle*" too much.

In connection with Dr. Gay's paper, it may be well to say that chloroform was used in puerperal convulsions some time prior to the report of his case; and we have no doubt he will be glad to relinquish all claims to priority, since, in vol. iv, page 206, (July 18th, 1848,) of the *Buffalo Medical Journal*, will be found full report of a successful use of it by Prof. James P. White, but without any claim to priority in its use.

